# Overexpression of TSC-22 (transforming growth factor-β-stimulated clone-22) causes marked obesity, splenic abnormality and B cell lymphoma in transgenic mice

**DOI:** 10.18632/oncotarget.7308

**Published:** 2016-02-10

**Authors:** Daisuke Uchida, Hitoshi Kawamata, Fumie Omotehara, Yoshihiro Miwa, Hideki Horiuchi, Tadashi Furihata, Masatsugu Tachibana, Takahiro Fujimori

**Affiliations:** ^1^ Department of Oral and Maxillofacial Surgery, Dokkyo Medical University School of Medicine, Mibu, Shimo-tsuga, Tochigi, Japan; ^2^ Department of Surgical and Molecular Pathology, Dokkyo Medical University School of Medicine, Mibu, Shimo-tsuga, Tochigi, Japan; ^3^ Department of Pharmacology, Institute of Basic Medical Sciences, The University of Tsukuba, Tsukuba, Ibaragi, Japan

**Keywords:** TSC-22, Tg-mice, obesity, splenic abnormality, B cell lymphoma

## Abstract

In this study, we generated transgenic (Tg) mice, which overexpressed transforming growth factor (TGF)-β stimulated clone-22 (TSC-22), and investigate the functional role of TSC-22 on their development and pathogenesis. We obtained 13 Tg-founders (two mice from C57BL6/J and 11 mice from BDF1). Three of 13 Tg-founders were sterile, and the remaining Tg-founders also could generate only a limited number of the F1 generation. We obtained 32 Tg-F1 mice. Most of the Tg-mice showed marked obesity. Histopathological examination could be performed on 31 Tg-mice; seventeen mice died by some disease in their entire life and 14 mice were killed for examination. Most of the Tg-mice examined showed splenic abnormality, in which marked increase of the megakaryocytes, unclearness of the margin of the red pulp and the white pulp, and the enlargement of the white pulp was observed. B cell lymphoma was developed in 10 (71%) of 14 disease-died F1 mice. These results indicate that constitutive over-expression of TSC-22 might disturb the normal embryogenesis and the normal lipid metabolism, and induce the oncogenic differentiation of hematopoietic cells.

## INTRODUCTION

*Transforming growth factor (TGF)-β stimulated clone-22* (*TSC-22)* was originally isolated as a TGF-β-inducible gene in mice osteoblastic cells, MC3T3E1 [1]. Then, *TSC-22* was shown to encode a putative transcriptional regulator containing a leucine zipper-like structure [1]. Subsequently, *TSC-22* was demonstrated to be up-regulated by many different stimuli such as TPA, choleratoxin, dexamethasone [1], follicle-stimulating hormone [2], tumor necrosis factor α, interferon-γ, interleukin-1β, lipopolysaccharide [3], progesterone [4], and epidermal growth factor (EGF) [5]. We identified human *TSC-22* as an anti-cancer drug (Vesnarinone)-inducible gene in a human salivary gland cancer cell line, TYS [6, 7]. Recently we [8] and other investigators [9] demonstrated that upregulation of TSC-22 mediated by TGF-β can be a post-transcriptional regulation via some microRNAs. TSC-22 contained leucine zipper motif and TSC-box but did not have a classical DNA-binding domain as bZip or bHLH-Zip families did. Therefore, TSC-22 was hypothesized to act as a transcritptional regulator by binding other leucine zipper containing transcription factors. Ohta *et al.* reported TSC-22 as a transcription factor for C-type natriuretic peptide gene [3]. However, we and Kester *et al.* recently reported that TSC-22 acted as a transcriptional enhancer [10] or repressor [11] when fused to the DNA binding domain of yeast transcription factor GAL4. Thus, the mechanism underlying the transcriptional up-regulation of TSC-22 remained obscure.

Concerning the role of TSC-22 on cell growth and differentiation, and tumorigenesis, we reported that TSC-22 negatively regulated the growth of TYS cells [6], and that downregulation of TSC-22 in TYS cells played a major role in the salivary gland tumorigenesis [7]. Subsequently, we reported that overexpression of TSC-22 enhanced chemosensitivity and radiation-sensitivity by inducing apoptosis in the cancer cells [12–14]. Recently, Yu *et al.* and Kato *et al.* reported the characteristics of the TSC-22 deficient mice [9, 15]. They concluded that TSC-22 had a tumor suppressor function in T or natural killer large granular lymphocyte leukemia, and carcinogen–induced liver tumor.

On the other hand, several investigators clarified the crucial role of TSC-22 on the development of Drosophila and mice [16–20]. Treisman *et al.* [16], Kania *et al*. [17] and Dobens *et al*. [18] found that Drosophila TSC-22 gene, *shortsighted* or *bunched*, was essential for the Drosophila development, and that Drosophila TSC-22 gene was an effector gene which could integrate multiple extracellular signals. Furthermore, Dohrmann *et al.* [19] and Kester *et al*. [20] demonstrated that, during mouse embryogenesis, TSC-22 was upregulated at sites of epithelial-mesenchymal interaction and expressed in many neural crest-derived tissues.

In this study, we generated transgenic mice, which overexpressed TSC-22 in most of the cells in the entire body, and investigated the functional role of TSC-22 on the development and pathogenesis of mice.

## RESULTS

### Expression of EGFP-TSC-22 fusion proteins in TYS cells from the transgenic cassette

**Figure 1:**
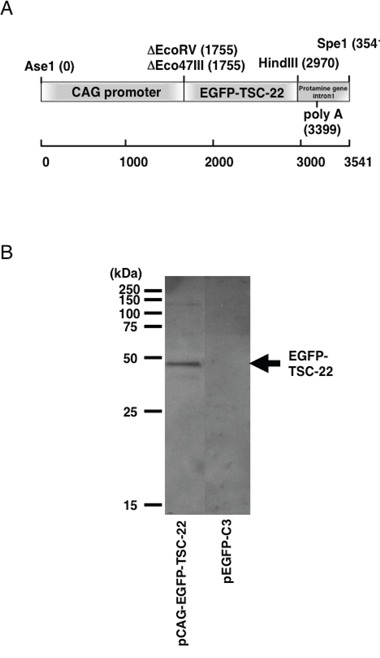
Structure and expression of the EGFP-TSC-22 fusion gene **A.** Schematic structure of the TSC-22 transgenic cassette. Transgenic cassette contains human TSC-22 fragment fused to EGFP under the transcriptional control of CAG promoter. The human TSC-22-EGFP fusion gene was followed by the first intron of Protamine gene containing the poly-adenylation signal. **B.** Expression of EGFP-TSC-22 fusion proteins from the transgenic cassette in a human salivary gland cell line, TYS cells. TYS cells were transfected with the transgenic cassette. Forty-eight hours after transfection, cell lysates were prepared from the transfectants, and the expression of EGFP-TSC-22 fusion protein (45 kDa) was examined by Western blotting.

We transiently transfected TYS cells with the transgenic cassette containing *EGFP-TSC-22* fusion gene (Figure 1A). GFP fluorescence was observed only in the cytoplasm but not in the nucleus in the EGFP-TSC-22-transfectants (data not shown). As we have already reported [10, 13], TSC-22 protein contains a nuclear export signal (NES), and localizes to cytoplasm in the live cells. By Western blotting (Figure 1B), we confirmed the expression of EGFP-TSC-22 fusion protein (45 kDa) in the EGFP-TSC-22 transfectants, but not in the control transfectants (pEGFP-C3).

### Generation of TSC-22 transgenic mice expressing EGFP-TSC-22 fusion protein

We transferred 134 DNA-injected embryos to C57BL6/J pseudopregnant foster mothers; however, we obtained only 21 mice (Table 1). Moreover, only 2 mice (one male and one female) were positive for TSC-22 transgene in the 21 born mice (Table 1). The ratio of the born mice per transferred embryos (15.7%) and the ratio of the transgene-positive mice per born mice (9.5%) were much lower than the ratio in other gene transferred mice in this institute. Then, we changed the mouse strain from in-bred (C57BL6/J) to hybrid (BDF1). We transferred more than 300 DNA-injected embryos to BDF1 pseudopregnant foster mothers, and obtained 121 mice. Eleven mice (seven male and four female) were positive for TSC-22 transgene in the 121 born mice (9.0%).

**Table 1:** Number of transgene-positive mice

	No. of transferred DNA-injected embryos	No. of Tg-positive mice/No. of born mice	(% Tg-positive mice)
C57BL6/J	134	2 (♂: 1, ♀: 1)/21	9.5%
BDF1	>300	11 (♂: 7, ♀: 4)/121	9.0%

**Figure 2:**
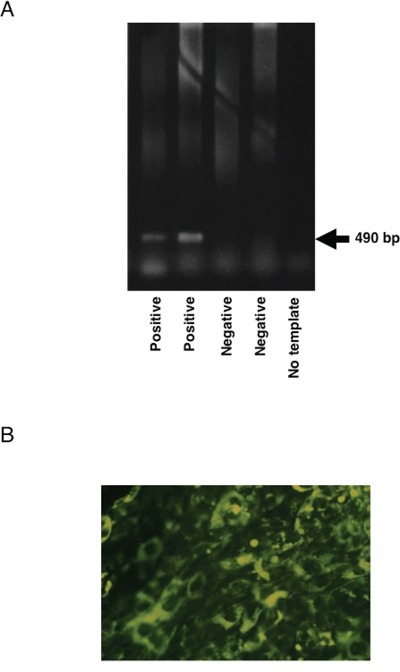
Generation of EGFP-TSC-22 transgenic mice **A.** Genotyping of transgenic mice. Genotyping of transgenic mice were performed by the PCR amplification of the EGFP-TSC-22 gene. Genomic DNA was extracted from ear biopsies. The 490 bp of human EGFP-TSC-22 fusion gene in the transgenic cassette was amplified. **B.** Expression of EGFP-TSC-22 fusion protein in the transgenic founders. The expression of the EGFP-TSC-22 fusion protein was confirmed in the cultured keratinocytes obtained from the transgene-positive mice. GFP fluorescence (EGFP-TSC-22 fusion protein) was clearly observed in the cytoplasm but not in the nucleus in the keratinocytes.

Genotyping of the transgenic mice were performed by the PCR amplification of the EGFP-TSC-22 gene. Transgene-positive mice produced 490 bp band by the PCR amplification as shown in Figure 2A. The expression of mRNA from the EGFP-TSC-22 gene in the transgene-positive mice was examined by RT-PCR as described above. Transgene-positive mice certainly expressed EGFP-TSC-22 mRNA at very high level (data not shown). Furthermore, the expression of the EGFP-TSC-22 fusion protein was confirmed in the cultured keratinocytes obtained from the transgene-positive mice. GFP fluorescence (EGFP-TSC-22 fusion protein) was clearly observed in the cytoplasm but not in the nucleus in the keratinocytes (Figure 2B).

### Breeding of transgenic mice

Number of the transgene-positive F1 mice in each TSC-22 transgenic founder was shown in Table 2. Female transgenic founder in C57BL/6 strain bore total 22 pups at four times delivery; however, none of the pups were transgene positive. Moreover, male transgenic founder in C57BL/6 strain was sterile. Transgenic founders in BDF1 strain produced several F1 transgene-positive mice. Number of the pups at one delivery was extremely limited at the range from 0 to 11 (average 6) both in male and female BDF1 transgenic founder. One male and one female BDF1 transgenic founders were sterile. Furthermore, the ratio of the transgene-positive F1 mice per born mice was extremely low. We obtained 32 transgene-positive F1 mice, 12 male and 20 female.

**Table 2:** Number of transgene-positive F1 mice in each TSC-22 transgenic founder mice

Founders	1^st^ delivery	2^nd^ delivery	3^rd^ delivery	4^th^ delivery	5^th^ delivery
P♀1 (C57BL/6)	0/6^a^ (♂3, ♀3)	0/9 (♂4, ♀5)	0/7 (♂5, ♀2)		
P♀2 (BDF1)	0/4 (♂2, ♀2)	0/2 (♂2, ♀0)	2 (♂1, ♀1)/4 (♂1, ♀3)	1 (♀1)/2	
P♀3 (BDF1)	sterile				
P♀4 (BDF1)	0/1 (♂1)	0/7 (♂3, ♀4)	0/7	3 (♂2, ♀1)/7	
P♀5 (BDF1)	3 (♀3)/10 (♂3, ♀7)	2 (♂1, ♀1)/9 (♂5, ♀4)	0/2	0/7 (♂1, ♀2)	
P♂1 (C57BL/6)	sterile				
P♂2 (BDF1)	sterile				
P♂3 (BDF1)	0/7 (♂4, ♀3)	1 (♀1)/11 (♂6, ♀5)	2 (♂1, ♀1)/9		
P♂4 (BDF1)	0/4 (♂2, ♀8)	1 (♂1)/7	0/7 (♂5, ♀2)	3 (♀3)/7	
P♂5 (BDF1)	0/1 (♂1)	0/8 (♂5, ♀3)	1 (♀1)/9		
P♂6 (BDF1)	0/4 (♂2, ♀2)	0/4 (♂5, ♀3)	0/4 (♂1, ♀5)	0/6	
P♂7 (BDF1)	2 (♀2)/8 (♂1, ♀7)	0/9 (♂?, ♀?)	4 (♂4)/10	5 (♀5)/10	
P♂8 (BDF1)	0/1 (♂1)	0/5 (♂3, ♀2)	1 (♂1)/9	0/10 (♂1, ♀9)	1 (♂1)/9

a Number of transgene-positive mice/number of born mice.

### Overexpression of TSC-22 causes marked obesity in mice

**Figure 3:**
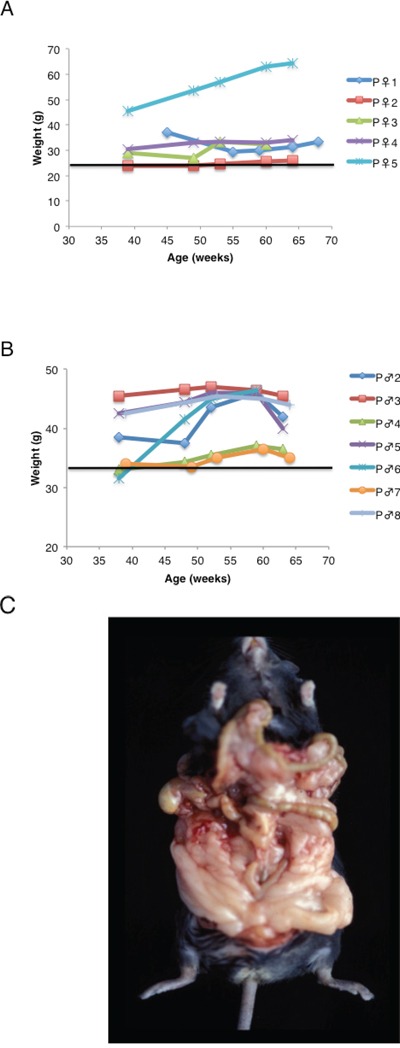
Overexpression of TSC-22 causes marked obesity in mice Most of the TSC-22 transgenic mice showed marked obesity both in female **A.** and male **B.** The accumulation of the subcutaneous fat tissue and the intra-abdominal fat tissue was observed. Body weight of the transgenic mice was much higher than that of wild type C57BL6 or DBA2. Horizontal bars in each graph show the mean weight of adult mice. **C.** The body weight of the P♀5 (BDF1) was over 60 g, which was twice as that of wild type. Most of the F1 mice inherited the phenotype, obesity from the founder.

Most of the TSC-22 transgenic mice showed marked obesity. As shown in Figure 3AB, body weight of the transgenic mice was much higher than that of wild type C57BL6 or BDF1 (data was obtained from Clea Japan web site; http://www.clea-japan.com/animalpege/a_1/e_02.html or http://www.clea-japan.com/animalpege/a_1/f_02.html). Especially, the body weight of the P♀5 (BDF1) was over 60 g, which was twice as that of wild type. The accumulation of the subcutaneous fat tissue and the intra-abdominal fat tissue was observed (Figure 3C). Most of the F1 mice inherited the phenotype, obesity from the founder.

### Histopathological abnormality and a principal cause of death of TSC-22 transgenic mice

**Figure 4:**
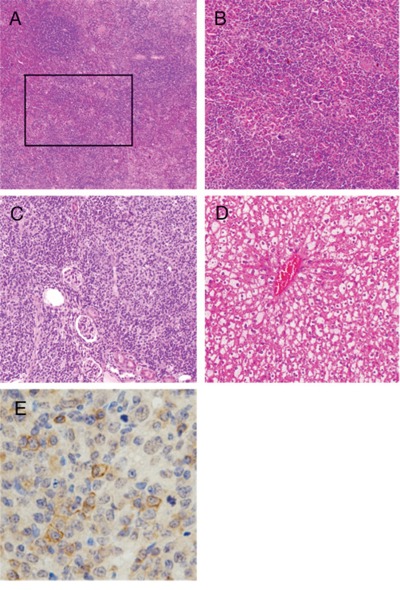
Histopathological abnormality of TSC-22 transgenic mice **A, B.** Most of the transgenic mice showed splenic abnormality, in which marked increase of the megakaryocytes, unclearness of the margin of the red pulp and the white pulp, and the enlargement of the white pulp was observed. **C.** Malignant lymphoma was developed in ten (71%) of 14 disease-died F1 mice. Malignant lymphoma invaded into the kidney. **D.** Most of the transgenic mice with obesity showed the marked fatty-degeneration of hepatocytes. **E.** Immunohistochemistry by a monoclonal antibody HM57 clearly showed the expression of CD79α on the cell surface.

Nine of 13 founder TSC-22 transgenic mice (Table 3) and 22 of 32 F1 TSC-22 transgenic mice were available for histopathological examination (Table 4). In 31 transgenic mice examined, 17 mice died by some disease in their entire life and 14 mice were killed for examination. Most of the transgenic mice showed splenic abnormality, in which marked increase of the megakaryocytes, unclearness of the margin of the red pulp and the white pulp, and the enlargement of the white pulp was observed (Figure 4A, 4B). Lymphoma (Figure 4C) was developed in ten (71%) of 14 F1 mice died by some disease. Hemangioma in the liver or spleen was observed in five (29%) of the 17 mice. Most of the transgenic mice with obesity showed the marked fatty-degeneration of hepatocytes (Figure 4D). Pulmonary emphysema was developed in 4 of the 17 mice, and adenocarcinoma of the lung was observed in 3 of the 14 F1 mice. Immunohistochemistry by a monoclonal antibody HM57 clearly showed the expression of CD79α on the lymphoma cells (Figure 4E), then all of the lymphoma was confirmed to be B cell lymphoma.

**Table 3:** Possible cause of death and histopathological features of TSC-22 transgenic founder mice

Founder mice	Died or killed	Possible cause of death	Histopathological features
P♀1 (C57BL/6)	Died at 2 years 1 month	Renal failure	Adenoma in lung, fibrosis of nephron, skin and heart, fatty liver, splenic abnormality
P♀2 (BDF1)	Died at 2 years 1 month	Not determined	Not available
P♀3 (BDF1)	Died at 2 years	Not determined	Not available
P♀4 (BDF1)	Killed at 2 years	Killed	No remarkable change
P♀5 (BDF1)	Killed at 1 year 3 months	Killed	Fatty liver, splenic abnormality
P♂1 (C57BL/6)	Died at 3 months	Not determined	Not available
P♂2 (BDF1)	Killed at 1 year 3 months	Killed	Fatty liver
P♂3 (BDF1)	Died at 1 years 5 months	Rupture of hemangioma	Hemanginoma in spleen
P♂4 (BDF1)	Killed at 2 years	Killed	Fatty liver, splenic abnormality, renal cysts
P♂5 (BDF1)	Died at 1 years 2 months	Not determined	Not available
P♂6 (BDF1)	Killed at 2 year	Killed	No remarkable change
P♂7 (BDF1)	Killed at 1 year	Killed	Fatty liver, splenic abnormality
P♂8 (BDF1)	Died at 1 years 10 months	Pulmonary emphysema	Severe pulmonary emphysema

**Table 4:** Possible cause of death and histopathological features of TSC-22 transgenic F1 mice

F1 mice	Died or killed	Possible cause of death	Histopathological features	Reproductive function
3#8♀ (P♀2)	Died at 2 years 4 months	Lung cancer	Adenocarcinoma of lung with mediastinal metastasis	Not mated
3#5♂ (P♀2)	Died at 1 month	Not determined	Not available	Not mated
4#2♀ (P♀2)	Died	Not determined	Not available	Sterile
4#4♀(P♀4)	Died at 2 year 2 months	Malignant lymphoma	Multiple metastasis of malignant lymphoma	Not mated
4#1♂ (P♀4)	Killed at 7 months	Killed	Fibrosis of skin, splenic abnormality	Not mated
4#5♂ (P♀4)	Died at 2 years 2 months	Rupture of hemangioma	Hemanginoma in liver	Fertile
1#5♀ (P♀5)	Killed at 1 year 2 months	Killed	Fatty liver, splenic abnormality	Not mated
1#6♀ (P♀5)	Died	Not determined	Not available	Sterile
1#7♀ (P♀5)	Killed at 1 year 2 months	Killed	Fatty liver, splenic abnormality	Not mated
2#4♂ (P♀5)	Died	Not determined	Not available	Fertile
2#7♀ (P♀5)	Died at 2 years 8 months	Malignant lymphoma	Multiple metastasis of malignant lymphoma	Sterile
2#10♀ (P♂3)	Died at 2 years 5 months	Rupture of hemangioma	Hemanginoma in liver, pulmonary emphysema, splenic abnormality	Sterile
3#7♀ (P♂3)	Died at 2 years 8 months	Malignant lymphoma	Multiple metastasis of malignant lymphoma, renal cysts adenocarcinoma of lung, hemanginoma in liver	Sterile
3#6♂ (P♂3)	Died at 2 years	Not determined	Not available	Sterile
2#2♂ (P♂4)	Killed at 7 months	Killed	Fibrosis of skin, splenic abnormality	Not mated
4#5♀ (P♂4)	Died	Not determined	Not available	Still birth once
4#6♀ (P♂4)	Died at 1 year 2 months	Not determined	Not available	Not mated
4#7♀ (P♂4)	Died at 2 years	Malignant lymphoma	Multiple metastasis of malignant lymphoma	Still birth three times
3#3♀ (P♂5)	Died at 1 year 10 months	Not determined	Not available	Sterile
1#6♀ (P♂7)	Died at 2 years 4 months	Not determined	Not available	Sterile
1#7♀ (P♂7)	Died at 2 years 5 months	Malignant lymphoma	Multiple metastasis of malignant lymphoma	Not mated
3#4♂ (P♂7)	Died at 1 year 5 months	Pulmonary emphysema	Severe pulmonary emphysema, sclerosis of heart	Not mated
3#9♂ (P♂7)	Killed at 2 years	Killed	Fatty liver, splenic abnormality	Not mated
3#10♂ (P♂7)	Killed at 2 years	Killed	Adenocarcinoma of lung	Not mated
3#11♂ (P♂7)	Killed at 2 years	Killed	Fatty liver, splenic abnormality	Not mated
4#3♀ (P♂7)	Killed at 1 year 11months	Killed	Fatty liver, splenic abnormality	Sterile
4#5♀ (P♂7)	Died	Not determined	Not available	Sterile
4#6♀ (P♂7)	Died at 1 year 11 months	Malignant lymphoma	Multiple metastasis of malignant lymphoma	Sterile
4#8♀ (P♂7)	Died at 1 year 11months	Malignant lymphoma	Multiple metastasis of malignant lymphoma	Sterile
4#10♀ (P♂7)	Died at 1 year 7 months	Malignant lymphoma	Multiple metastasis of malignant lymphoma	Sterile
3#2♂ (P♂8)	Died at 2 year 10months	Malignant lymphoma	Multiple metastasis of malignant lymphoma	Not mated
5#7♂ (P♂8)	Died at 1 year 5 months	Rupture of hemangioma, malignant lymphoma	Hemanginoma in liver, pulmonary emphysema, multiple metastasis of malignant lymphoma	Fertile

### Blood analysis of the TSC-22 transgenic mice

The average and standard deviation of the number of red blood cells, white blood cells, and platelets in the blood of 20 transgenic mice were 713 ± 121 ×10^4^/μl, 6226 ± 2951/μl, 90 ± 44 ×10^4^/μl, respectively. The average and standard deviation of serum glucose levels in 22 transgenic mice was 161 ± 38 mg/dl. These data in the transgenic mice were almost the same as that of wild type BDF1 (data was obtained from Clea Japan web site; http://www.clea-japan.com/animalpege/a_1/f_03.html).

## DISCUSSION

Several investigators have demonstrated that TSC-22 regulates cell growth and differentiation as well as cell death [1–4, 6, 7, 21], and that TSC-22 is required for the embryonic development [16–20]. Expression of TSC-22 mRNA was reported to be detectable at 6.5 day embryos ubiquitously [19]. TSC-22 was upregulated at the site of epithelial-mesenchymal interaction, such as limb bud, tooth germ, hair follicle, kidney, lung, and pancreas. TSC-22 was also upregulated at neural crest derived tissues, heart, otic and optic vesicle, and cartilage and bone forming region throughout the embryo [19, 20]. In the present study, number of the pups at one delivery was extremely limited, and the ratio of the transgene-positive F1 mice per born mice was extremely low. Although these reasons remain unclear, artificial over-expression of TSC-22 during embryogenesis might inhibit several epithelial-mesenchymal interactions and disturb normal development of the embryos in the transgenic mouse. More detail histopathological examinations concerning embryogenesis in this transgenic mouse must be conducted.

Jay *et al.* reported the expression of TSC-22 in adult mouse [22]. TSC-22 mRNA was highly expressed at ovary, small intestine, colon, heart, and brain, and moderately expressed at thymus, prostate, testis, placenta, skeletal muscle, and pancreas, and faintly expressed at spleen, lung, liver, and kidney in adult mouse. However, TSC-22 was not expressed in leukocytes in adult mouse. Interestingly, abnormal organs in our transgenic mouse were almost corresponding to the organs with limited TSC-22 expression in adult mouse. Constitutive over-expression of the TSC-22 in the organs with limited TSC-22 expression in adult mouse might inhibit the growth of the cells and/or cause the differentiation abnormality of the cells. Yu *et al.* [15] and Nakamura *et al.* [23] generated the TSC-22 knock-out mice, and they showed its crucial role on the proliferation and the repopulation efficacy of hematopoietic precursor cells, and on the oncogenic Ras/Raf signaling, respectively. However, Yu *et al.* described that TSC-22 knock-out mice showed slight weight decrease of kidneys and hearts, and Nakamura *et al.* mentioned that TSC-22 knock-out mice were born at the expected Mendelian ratio and showed no obvious abnormality. In the TSC-22 knock-out mice, other TSC-22 families might compensate the role TSC-22 on the development of the mouse.

Some investigators reported the implication of TSC-22 on type 2 Diabetes or lipid metabolism [9, 24–26]. In our transgenic mice, blood glucose level was not elevated, and renal fibrosis was not observed. At present, we could not clearly explain why our transgenic mice became fat, we should examine the food and water intake, eliminative behavior, and motor behavior in the metabolic gage.

From the beginning of the discovery of TSC-22 [1, 2, 6, 7], TSC-22 was considered as a tumor suppressor, a differentiation induce, or a negative growth regulator. Further consolidation data was continuously published in several journals [27–34]. However, in our transgenic mice, frequent development of B cell lymphoma was observed. Although Yamate *et al.* reported that malignant lymphoma was developed in wild type BDF1 mice allowed to live out their life-span [35], in our experiment, B cell lymphoma was developed in 59% of the disease-died transgenic mice. Most of the Tg-mice examined showed splenic abnormality, such as unclearness of the margin of the red pulp and the white pulp, and the enlargement of the white pulp. These observations suggested that several abnormalities might occur in the lymphocytes of the transgenic mice after survival of the growth suppression, the induction of apoptosis and the differentiation during the embryogenesis and the development. Therefore, lymphocytes in the born-TSC-22 transgenic mice might be susceptible for the development of lymphoma. TSC-22 was previously reported to inhibit the development of several hematopoietic malignancies [15, 31], and enhance the differentiation of the several hematopoietic cells [36]. Therefore, our transgenic mouse might be useful for examining the pathogenesis of hematopoietic malignancy as well as the metabolic abnormality and obesity.

## MATERIALS AND METHODS

### Ethics statement

Investigation has been conducted in accordance with the ethical standards and according to the Declaration of Helsinki and according to national and international guidelines and has been approved by the authors' institutional review board.

### Construction of the transgenic cassette

Transgenic cassette (Figure 1A) was constructed as follows: pCAGPrm was digested with EcoRV (Takara Biomedicals, Kusatsu, Japan) and BamH1 (Takara Biomedicals). The human TSC-22 cDNA fragment fused to enhanced green fluorescent protein (EGFP) cDNA was excised from pEGFP-TSC-22FL [10] by digesting with Eco47III (Takara Biomedicals) and BamH1 (Takara Biomedicals). Then, the human TSC-22 fragment fused to EGFP was ligated to the prepared cloning site of pCAGPrm, and pCAGPrm-TSC-22FL was generated. The direction of the ligated fragment from the promoter and the sequence of the ligated site in the plasmid were confirmed by sequencing analysis (Amersham Pharmacia Biotech., Uppsala, Sweden; Shimadzu DSQ-500 DNA sequencer, Shimadzu, Kyoto, Japan). pCAGPrm-TSC-22FL contained human TSC-22 fragment fused to EGFP under the transcriptional control of CAG promoter, which was composed of chicken actin promoter and cytomegalovirus immediate early promoter (CMV-IE). It is well known that the CAG promoter is one of the strongest promoters in the wide variety of the mammalian cells, and induces constitutive expression of the regulating gene [37]. The human TSC-22 fragment fused to EGFP was followed by the first intron of Protamine gene containing the poly-adenylation signal. Then, transgenic cassette (Figure 1A) was excised from pCAGPrm-TSC-22FL by digesting with Ase1 (Takara Biomedicals) and Spe1 (Takara Biomedicals).

### Determination of the expression of EGFP-TSC-22 fusion proteins from the transgenic cassette in TYS cells

A human salivary gland cell line, TYS cells [38] (5 × 10^5^ cells/dish) were seeded in 60-mm culture dishes (Falcon; Becton Dickinson Labware, Lincoln Park, NJ) in DMEM (Life Technologies, Inc., Gaithersburg, MD) supplemented with 10% FCS (Bio-Whittaker, Walkersville, MD), 100 μg/ml streptomycin, 100 U/ml penicillin (Life Technologies, Inc.), and 0.25 μg/ml amphotericin B (Life Technologies, Inc.) in a humidified atmosphere of 95% air and 5% CO_2_ at 37°C. Twenty-four h later, the cells were transfected with 1 μg of transgenic cassette using Superfect regent (QIAGEN, Hilden, Germany).

The expression of EGFP-TSC-22 fusion protein from the transgenic cassette was first examined in cultured-TYS cells by observing the GFP fluorescence. Forty-eight hours after transfection, GFP fluorescence was observed by a fluorescent-microscopy excited by 495 nm blue light on the transfectants (Nikon, Tokyo, Japan). Subsequently, expression of the fusion protein was confirmed by Western blotting using an anti-TSC-22 antibody [7]. Cell lysates were prepared from the transfectants by use of the cell lysis buffer [50 mM HEPES (pH 7.5) containing 150 mM NaCl, 1% Triton X-100, 1.5 mM MgCl_2_, 1 mM EDTA, 10 mM sodium pyrophosphate, 100 mM sodium orthovanadate, 100 mM NaF, 100 mM p-nitrophenyl phosphate, 5 U/ml aprotinin, and 1 mM phenylmethylsulfonyl fluoride]. The protein concentrations of the samples were determined by Bio-Rad protein assay (Bio-Rad, Hercules, CA). One hundred microgram of protein samples were electrophoresed on SDS-polyacrylamide gel. Proteins from gels were transferred to nitrocellulose (Bio-Rad), and the membrane was incubated with the affinity purified anti-TSC-22 antibody, and an Amersham ECL kit (Amersham Pharmacia Biotech.).

### Generation of TSC-22 transgenic mice

The transgenic founders were generated at Laboratory Animal Resource Center, University of Tsukuba under the control of Animal Care and Use Committee, University of Tsukuba. The transgenic cassette was injected into pronucleus of one cell-stage of embryos obtained from C57BL6/J (Clea Japan, Tokyo, Japan) or BDF1 (Clea Japan). The embryos were transferred to pseudopregnant foster mothers. After obtaining pups, the TSC-22 transgenic founders were distinguished by amplifying the human TSC-22-GFP fusion gene in the transgenic cassette using polymerase chain reaction (PCR) method. Genomic DNA was extracted from ear biopsies. The ear biopsies were incubated in 200 μl of the extracting buffer [10 mM Tris-HCl (pH 8.3), 50 mM KCl, 2.5 mM MgCl2, 0.1 mg/ml Gelatin, 0.45% Nonidet P-40, 0.45% Tween 20, 0.2 mg/ml Proteinase K] for 8 hours at 56°C, and boiled for 15 minutes. Then, the supernatants were obtained by centrifugation at 15,000 rpm for 10 minutes at 15°C. Subsequently, one microliter of the supernatants, which contained genomic DNA from the mice, was subjected to PCR amplification. The 490 bp of human TSC-22-GFP fusion gene in the transgenic cassette was amplified by a pair of primers (5′-CAA CAT CCT GGG GCA CAA GC-3′ as an up-stream primer, and 5′-CCA CAC TTG CAC CAG AGG AG-3′ as a down stream primer). PCR was performed as follows: the final concentration of dNTPs and primers in the reaction mixture were 200 μM and 1 μM, respectively. Taq DNA polymerase (Takara Biomedicals) was added to the mixture at a final concentration of 0.05 U/μ1, and the reaction was carried out in a Takara Thermal Cycler MP (Takara Biomedicals) under the following conditions: 94°C for 3 min and then 94°C for 1 min, 62°C for 1.5 min, 72°C for 2.5 min for 25 cycles, and extension at 72°C for 4 min.

### Expression of TSC-22-GFP fusion protein in the transgenic founders

Total RNA was extracted from tail biopsy in the transgenic founders by use of ISOGEN RNA extracting mixture (Nippon Gene, Toyama, Japan). The RNA was reverse-transcribed by Moloney murine leukemia virus (Life Technologies, Inc.) at 42°C for 60 min using random primer (5 μM; Life Technologies, Inc.) in 20 μ1 of the reaction mixture. Subsequently, one microliter of the products was subjected to PCR amplification. PCR was performed under the same condition as described above.

Primary cultured-keratinocytes were obtained from the tail biopsy following a standard protocol [39]. Keratinocytes were seeded on the cover glass (Muto pure chemicals Co., Tokyo, Japan) in 35-mm culture dishes. Twenty-four hour after inoculation, GFP fluorescence in the cultured cells was observed by the fluorescent-microscopy as described above.

### Breeding of transgenic mice

All mice were housed and bred in Laboratory Animal Research Center, Dokkyo Medical University School of Medicine with controlled photoperiods (14 hours light and 10 hours darkness), temperature, and humidity. The animals were maintained and treated in accordance with the policies of Dokkyo Medical University′s Animal Care and Use Committee. Mice were sacrificed by exsanguination under sodium pentobarbital anesthesia. Founder mice of TSC-22 transgenic mice were crossed with wild type C57BL6/J mice or wild type BDF1 mice to generate F1 generation. To assess fertility, pairs of the transgenic mice and wild type partners were housed in individual cages and numbers of pups per litter were recorded. Genotyping of the pups was performed by PCR method as described above. Body weight of the transgenic mice was measured at least once a week. Furthermore, behavior of the transgenic mice was carefully observed.

### Determination of the expression of TSC-22-GFP mRNA in the F1 transgenic mice

Total RNA was extracted from several organs including liver, pancreas, spleen, kidney, skin, skeletal muscle, brain, adipose tissue, esophagus, stomach, small intestine, colon, lung, and heart in the representative F1 transgenic mice by ISOGEN RNA extracting mixture (Nippon Gene, Toyama, Japan). Extracted total RNA was subjected to RT-PCR as described above.

### Histological examination

Several organs in the transgenic mice including liver, pancreas, spleen, kidney, adrenal gland, skin, skeletal muscle, brain, adipose tissue, esophagus, stomach, small intestine, colon, lung, salivary gland, spinal code, bone and heart were removed. All of the organs removed were fixed in a neutral aqueous phosphate-buffered 4% solution of formaldehyde and embedded in paraffin wax. The tissue was cut at 2-3 μm thickness and stained with hematoxylin and eosin.

### Immunohistochemistry

Sections (4-μm thick) were mounted on silane-coated glass slides, deparaffinized and rinsed. After antigen retrieval by microwave treatment in citric acid buffer (pH 6.0) for 95°C for 10 min, the sections were immersed in 0.3% hydrogen peroxide to block endogenous peroxidase activity. Subsequently, the sections were reacted without or with the anti-human CD79α primary antibody (clone HM57: Dako, Carpinteria, CA) for 1 h at room temperature. The sections were then incubated with the biotinylated secondary antibody in LSAB kit (Dako) for 15 min at room temperature. After washing with cold phosphate-buffered saline, streptavidin-peroxidase solution in LSAB kit was applied for 20 min. The sections were washed in cold phosphate-buffered saline, allowed to react with 3,3′-diaminobenzidine tetrahydrochloride solution and 0.03% hydrogen peroxide for 3 min at room temperature. The sections were finally counterstained with hematoxylin. When we replaced the primary antibody with phosphate-buffered saline, the clear staining in all of the cells was completely disappeared.

### Blood analysis of the TSC-22 transgenic mice

Number of red blood cells, white blood cells, and platelets in the blood of the transgenic mice were counted. Serum glucose levels in TSC-22 transgenic mice were also measured. Blood analyses were performed at SRL (Tokyo, Japan).
